# Remdesivir shifts circadian rhythmicity to eveningness; similar to the most prevalent chronotype in ADHD

**DOI:** 10.1007/s00702-021-02375-3

**Published:** 2021-07-17

**Authors:** Frank Faltraco, Denise Palm, Andrew Coogan, Adriana Uzoni, Isabell Duwe, Frederick Simon, Oliver Tucha, Johannes Thome

**Affiliations:** 1 Department of Psychiatry and Psychotherapy, University Medical Centre Rostock, Gehlsheimer Str. 20, 18147 Rostock, Germany; 2 Department of Psychology, Maynooth University, National University of Ireland, Maynooth, Ireland

**Keywords:** Remdesivir, Human dermal fibroblasts, Circadian rhythm

## Abstract

Circadian clocks control immunity and virus replication, as well as pharmacokinetics and efficacy therapeutics. The aim of this study was to investigate the extent of these relationships by measuring circadian gene expression in primary human-derived dermal fibroblast cultures (HDF) after remdesivir exposure. In the current study, we analysed circadian gene expression in a cohort of participants without a neuropsychiatric diagnosis. After ex vivo exposure to remdesivir to human dermal fibroblast (HDF) cultures and dexamethasone synchronization, the rhythmicity of circadian gene expression (*Clock, Bmal1, Per1-3, Cry1*) was analysed via qRT-PCR. In this study, D-MEQ scores indicated that participants without a neuropsychiatric diagnosis had no evening preference. Remdesivir leads to a slight phase-shift in *Clock, Per1* and *Per2*. Significant different expressions of *Bmal1* and *Per3* were detected after remdesivir exposure: *Bmal1* at ZT8 (*t*(22) = 3.26, *p* = 0.004), ZT24 (*t*(22) = − 2.66, *p* = 0.015), ZT28 (*t*(20) = − 2.14, *p* = 0.045) and *Per3* at ZT8 (*t*(22) = − 4.27, *p* < 0.001) and ZT12 (*t*(22) = − 2.61, *p* = 0.016). A significant difference between chronotype and circadian gene expression for *Bmal1, Cry1* and *Per3* was observed. The present study shows that remdesivir has an impact on circadian function. It is well known that the circadian rhythm effects sleep and, moreover, sleep quality. The results suggest that remdesivir medication may alter sleep quality in participants without a neuropsychiatric diagnosis and shifts chronotype to eveningness; similar as prevalent in ADHD.

## Introduction

Sleep plays a fundamental role in mental and physical health. Adequate sleep duration is essential for coping with major life events such as the COVID-19 pandemic caused by the severe acute respiratory syndrome coronavirus 2 (SARS-CoV-2) infection. Daily rhythms regulate the pharmacokinetics and pharmacodynamics of several therapeutic measures (Smolensky et al. 2017). Therefore, to obtain the best possible clinical outcomes circadian rhythms should be considered when designing and administering possible therapeutic interventions against COVID-19. Most of these are antivirals which are directed against intracellular or even nuclear targets.

The core circadian rhythm is regulated by the clock genes represented by *circadian locomotor output cycles kaput* (*Clock*), *brain and muscle Arnt-like protein-1* (*Bmal1*), *period* (*Per*) and *cryptochrome* (*Cry*) genes. The master clock resides in the suprachiasmatic nucleus (SCN) of the hypothalamus and is entrained in peripheral cells (Schibler and Sassone-Corsi 2002), including fibroblasts (Balsalobre 1998). The molecular clock has also been characterised in immune cells, including T and B as well as dendritic cells (Bollinger et al.^2011^; Silver et al. 2012). Circadian rhythms influence sleep propensity, alertness and performance (Dijk and von Schantz 2005). Sleep is linked to immune functions (Besedovsky et al. 2012; Irwin 2019), in particular, to increased production of pro-inflammatory cytokines (Prather et al. 2009). Macrophages, important regulators of the immune response, exhibit robust circadian oscillation in gene expression, including genes responsible for pathogen recognition and cytokine secretion (Hayashi et al. 2007; Keller et al.^2009^). Circadian disruption, mimicking jet lag in mouse models, can greatly stimulate the release of interleukin-8 and other inflammatory cytokines induced by lipopolysaccharide, the most abundant components within the cell wall of *Gram-negative* bacteria (Castanon-Cervantes et al. 2010). An increase in immune response molecules is linked to clock-controlled enhanced sensitivity. Desynchronisation of the clock impairs immune function (Curtis et al. 2014). Peritoneal macrophages lacking *Bmal1* produce higher amounts of proinflammatory cytokine interleukin-6 in response to lipopolysaccharide after synchronisation in comparison to wild-type peritoneal macrophages (Gibbs et al.^2012^). *Bmal1* depletion leads to inflammatory reaction in myeloid cells (Nguyen et al. 2013), and herpes as well as influenza A virus infections are enhanced by *Bmal1* depletion (Edgar et al. 2016).

Sleep deprivation is linked to the alteration of the immune system. Partial sleep deprivation of fifty-two healthy volunteers for one night transiently impaired mitogen proliferation, decreased the human leukocyte antigen, isotype DR (HLA-DR) and upregulated the cluster of differentiation 14 (CD14), increasing susceptibility to respiratory infections (Wilder-Smith et al. 2013). Shorter sleep duration, measured behaviourally using actigraphy prior to rhinovirus exposure, was associated with increased susceptibility to the common cold (Prather et al. 2015).

The COVID-19 pandemic has produced significant stress, anxiety, and increased incidence of sleep disturbances (Morin et al. 2020). Psychiatric services are at risk of being overwhelmed at this time of unprecedented crisis, with an increased need to protect and defend the fundamental rights of patients (Thome et al. 2020a, b). Sleep disturbances are common among psychiatric disorders (Anderson and Bradley 2013). Silva et al. 2020, suggested that short sleep and sleep loss, which are common consequences of shift work, are associated with altered immune function, increasing the risk of COVID-19 (Silva et al. 2020). Shift-workers also report higher incidence and severity of respiratory infections (Archer et al. 2018; Archer and Oster 2015). Increased exposure to light at night may inhibit the production of melatonin, resulting in alterations in immune activity (Galano et al. 2018). Shift-workers show abnormal immune cell and cytokine levels (Loef et al. 2019). Vitale et al. (2020) assessed the influence of severe symptoms of COVID-19 infection on sleep quality in four patients during the sub-acute recovery stage of the disease. The authors observed lower sleep efficiency and immobility time and higher fragmentation index in patients with severe respiratory COVID-19 symptoms who were admitted to an intensive care unit (ICU) compared to patients with mild respiratory symptoms not requiring an ICU stay (Vitale et al. 2020). In contrast, Leone et al. reported overall improvement of sleep conditions in healthy subjects during lockdown (Leone et al. 2020). When comparing sleep duration, quality and timing, and social jetlag between control and lockdown conditions of 1021 subjects, the authors observed that participants slept longer and later during lockdown weekdays, and exhibited lower levels of social jetlag. Chronotype was, however, significantly delayed during the lockdown.

Based on behavioural manifestations of an individual's internal clock, one can display a morning, neutral or evening preference. A recent study suggests that chronotype plays an important role in the negative effects of home confinement of ADHD children during the COVID-19 outbreak (Cetin et al. 2020). Impaired sleep may occur as an adverse reaction of psychopharmacotherapy (Gahr et al. 2018).

Remdesivir is considered one of the most promising drug candidates for the treatment of COVID-19. Remdesivir, GS-5734 is a single diastereomer monophosphoramidate prodrug of a monophosphate nucleoside analog GS-441524. Intracellularly remdesivir is metabolised to the pharmacologically active analogous of adenosine triphosphate, GS-443902, that selectively inhibits viral RNA polymerases but not host RNA or DNA polymerases (Gordon et al. 2020). Remdesivir has a very short plasma half-life in non-human primates and mice (Ray and Reddy 2020), and has, in humans, shown 80–90% plasma protein binding (Tempestilli et al.^2020^).

Remdesivir demonstrated effectiveness against members of several virus families, including filoviruses and coronaviruses, such as SARS-CoV and Middle East respiratory syndrome coronavirus (MERS-CoV) (Sheahan 2020; Tchesnokov et al. 2019; Wang et al. 2020). In vitro testing has also shown that remdesivir can be used against COVID-19 (Augustin et al. 2020; Beigel et al. 2020; Grein et al. 2020). For individuals who are diagnosed early during illness with a high risk of hyperinflammation, 10- day remdesivir therapy shortens the time to recovery and reduces the risk of disease progression (Spinner et al. 2020). Furthermore, among patients with moderate COVID-19, 5-day remdesivir therapy is effective with the lowest risk of serious adverse events (Szarpak et al. 2020).

Several studies have evaluated the antiviral efficiency and effectiveness of remdesivir in cellular models. In human airway epithelial cells, remdesivir efficiently inhibited both MERS-CoV and SARS-CoV replication at 0.1, 1 and 10 µM concentrations. No cytotoxicity was observed at 10 μM remdesivir, the highest concentration tested (Sheahan et al. 2017). The Wuhan Institute of Virology analysed the antiviral efficiency of five FDA-approved drugs including ribavirin, penciclovir, nitazoxanide, nafamostat, and chloroquine, as well as antiviral drugs remdesivir and favipiravir, using Vero cells lines infected with SARS-CoV-2. Remdesivir potently blocked virus infection at low-micromolar concentrations, with EC50 value of 0.77 μM (Wang et al. 2020).

Glucocorticoid receptor agonist, dexamethasone may be useful in the short-term for severe, intubated, COVID-19 patients (Group et al. 2020). Glucocorticoids contribute to the synchronisation of the cell-autonomous clocks in peripheral cells and dexamethasone stimulates G1-S phase transition in human airway fibroblasts obtained from asthmatics (Dickmeis et al. 2007; Fouty et al. 2006).

Our research group successfully established a model based on patient-derived human dermal fibroblast (HDF) for the investigation of the circadian rhythm, particularly, circadian gene expression (Coogan et al. 2019). In a study, at the molecular level, we observed alterations in the expression of *Per2* and *Cry1* between HDF cultures obtained from ADHD individuals with no medication compared to HDF from healthy participants or medicated ADHD patients after 30 min dexamethasone synchronisation (Coogan et al. 2019). HDFs are an advantageous model to study the influence of drugs. The synchronization of the circadian system of fibroblasts can be achieved by several substances with different effects on circadian gene expression (Faltraco et al. 2020). HDF cultures are advantageous for in vitro functional models examining various cellular and molecular mechanisms (Kalman et al. 2016).

Depletion of cryptochrome genes in immortalised fibroblasts regulates the expression of proinflammatory cytokines (Van Linthout et al. 2014). Fibroblasts play a critical role in the switch of acute to chronic persistent inflammation (Narasimamurthy et al. 2012). Fibroblasts also produce inflammatory mediators such as toll-like receptors, antimicrobial peptides, proinflammatory cytokines, chemokines, and growth factors, as a response to microorganisms (Bautista-Hernandez et al. 2017). In a study, using normal human fibroblast lung cells as a model, a series of anti-HIV nucleosides and their fatty acyl derivatives were compared with remdesivir for antiviral activity against human coronavirus 229E (HCoV-229E). Only remdesivir was found to be potent, with an EC50 value of 0.07 µM and a therapeutic index of more than 28.6 µM (Parang et al. 2020).

The metabolism of drugs in some cases is extensive and missing knowledge of pharmacokinetics often results in misinterpretation of symptoms. Some drugs, e.g. psychostimulant medication, are known to influence sleep (Hvolby 2015). Considering that circadian clocks control immunity and virus replication, as well as the pharmacokinetics and efficacy of several therapeutics, an evaluable approach is to evaluate the effect of remdesivir on circadian genes. To address these issues, the circadian rhythms at the molecular level are investigated in participants without a neuropsychiatric diagnosis. Also, human dermal fibroblasts obtained from the study participants were exposed to remdesivir.

Based on the assumption of the effectiveness of remdesivir for the treatment of COVID-19 its influence on the expression of circadian genes is hypothesized. Therefore, in this study the influence of remdesivir on circadian rhythmicity is investigated using fibroblasts as in vitro model.

## Material and methods

### Participant selection criteria

Ethics approval for the conduct of the study, including obtaining human dermal biopsy samples, was given by the ethical review committee of Rostock University (Registration-number: A2013-159) and written consent was obtained from each study participant. The study was conducted according to the ethical guidelines of the Declaration of Helsinki.

In total, 12 volunteers without a neuropsychiatric diagnosis participating in the study were recruited via the Department of Psychiatry and Psychotherapy, University Medical Centre Rostock. Human dermal fibroblasts (HDF) were obtained from skin biopsies of the dorsal forearm area. Only adult individuals, able to give informed consent, were included. Shift-workers were excluded. The IQ of the participants were measured by using MWT (Multiple Choice Word Test). The chronotype of the participants were determined by the D-MEQ (Morning-Eveningness-Questionnaire, German Version). No special cognitive testing was implemented in the study.

The four manuscripts of this special issue dealing with circadian rhythmicity describe unique research questions (Faltraco et al. 2021a, b; Palm et al. 2021). Although some samples have been used for more than one research question, the overall sample composition differs from each other and thus is different for each study. Experiments differ substantially in their conditions, thus, they each investigate unique cellular biochemical pathways.

### Actigraphy

To obtain objective measures of participants’ sleep and circadian rhythm function, the rest–activity pattern of participants was recorded using wrist-worn actigraphs (Actiwatch 2, Philips Respironics, USA). Actigraphs were worn on the non-dominant wrist for a period of at least seven consecutive days. The recording interval of the device was set at 60-s epochs. Data occurring before the first and after the final midnight of each record were excluded, ensuring at least six complete days for each participant, with a complete weekend included in each record. The following sleep parameters were measured: mid-sleep on weekend (time of mid-sleep on free/weekend days), mid-sleep on weekdays (time of mid-sleep on work/weekdays), social jetlag (difference between mid-sleep on workdays and free days), sleep efficiency (difference between sleeping time and the time spending in bed), WASO (time spent in wakening after sleep onset) and total number of wake bouts (number of awakenings).

### Tissue isolation and fibroblast cell culture

Human dermal fibroblasts (HDF) were isolated and cultured as described previously (Takashima 1998). Fibroblasts were cultivated (37 °C, 5% CO_2_) in Dulbecco's Modified Eagle Medium DMEM (Gibco, Thermo Fisher, UK) /1 mg/ml Liberase TM (Roche, Germany) containing 100 units/ml penicillin, 100 µg/ml streptomycin (Gibco, Thermo Fisher, UK) and 10% fetal bovine serum FBS (Gibco, Thermo Fisher, UK).

### Measurement of cell viability

To determinate the optimal remdesivir concentration for human dermal fibroblasts, upon confluency of the respective primary fibroblast cell culture from each participant, cells were incubated with 0.00, 0.05, 0.50 and 1.00 µM remdesivir (MedChemExpress, Germany) for 24 h. Cell viability was measured using the Trypan Blue Exclusion Test (Strober 2015).

### Measurement of circadian gene expression

Upon confluency of the respective primary fibroblast cell culture from each participant, eight culture flask replicates were prepared and cells were incubated with 0.5 µM remdesivir (MedChemExpress, Germany). Cultures without remdesivir were used as a negative control. After 24-h of incubation, the cells were synchronised with 100 nm dexamethasone (Sigma-Aldrich, Germany) for 30 min. Samples were harvested every fourth hour after synchronisation for a period of 28-h in solution D (4.5 M guanidinium thiocyanate, 0.5% sodium-*N*-lauryl sarcosine, 25 mM tri-sodium citrate, 0.1 M betamercaptoethanol) and stored at − 80 °C. Total RNA was isolated and purified with RNeasy Plus Mini Kit (Qiagen, Germany), subjected to reverse transcription using the Superscript III First-Strand Synthesis System (Invitrogen, Germany) and gene expression of *Clock, Bmal1, Per1, Per2, Per3* and *Cry1* as well as and housekeeping genes (*Rpl13A, Rpl19A, GAPDH*) was measured by real-time quantitative reverse transcriptase-polymerase chain reaction (qRT-PCR) with CFX Connect™ Real-Time PCR Detection System (Biorad, Germany). All primers were purchased from Eurofins (Alameda, CA). The oligonucleotide sequences are presented in Table 1. The qRT-PCR was performed in 96-well 0.1-ml thin-wall PCR plates (Applied Biosystems) in the CFX Connect™ Real-Time PCR Detection System (Biorad, München, Germany) as previously described (Coogan et al. 2019).

**Table1:** Oligonucleotides for qRT-PCR to measure circadian gene expression

Gene	Forward primer (5ʹ–3ʹ)	Reverse primer (5ʹ–3ʹ)
*Clock*	CCAGCAGTTTCATGAGATGC	GAGGTCATTTCATAGCTGAGC
*Bmal1*	AAGGATGGCTGTTCAGCACATGA	CAAAAATCCATCTGCTGCCCTG
*Per1*	TGGGGACAACAGAACAGAGAA	AGGACACTCCTGCGACCA
*Per2*	GTATCCATTCATGCTGGGCT	TCGTTTGAACTGCGGTGAC
*Per3*	TCAGTGTTTGGTGGAAGGAA	TCTGGGTCAGCAGCTCTACA
*Cry1*	CACGAATCACAAACAGACGG	TACATCCTGGACCCCTGGT
*Rpl13a*	GCCAGAAATGTTGATGCCTT	AGATGGCGGAGGTGCAG
*Rpl19a*	GTGGCAAGAAGAAGGTCTGG	GCCCATCTTTGATGAGCTTC
*GAPDH*	GAAGGTGAAGGTCGGAGT	GAAGATGGTGATGGGATTTC

### Statistical methods

Circadian gene expression data were tested for significant circadian rhythmicity, using CircWave v. 1.4 software (generated by Dr. Roelof Hut; www.euclock.org) to determine the best-fitting linear harmonic regression with an assumed period of 24-h and with α set at 0.05. The center-of-gravity of each best-fitting waveform in CircWave was used as the circadian acrophase, and the associated estimation error was used as the SD. Inferential statistics were carried out in SPSS (IBM Corporation). qRT-PCR clock gene data were analysed via Student’s’ *t* Test. For all inferential tests, *p* < 0.05 was used to indicate a statistically significant groupwise difference. Sample sizes were calculated via GPower 3.1 software; for correlations the assumptions used were significance level of *α* = 0.05 and the power of 0.8 for one group with two measures (0.0 µM, 0.5 µM remdesivir). Although the research in this field is generally scarce, we assumed that the influence of remdesivir on the circadian gene expression will have an effect size *d*’ = 0.5, returning a required total sample size of 21. Taking into consideration an expected drop-out rate, *n* = 12 participants were allocated per each measure. One-way ANOVA was used to assess differences of circadian gene expression levels among chronotype groups. Associations between clock gene expression and chronotype obtained from the volunteers were studied by Spearman’s rank-order correlation. Data were analyzed via time-series statistics adequately powered by 12 samples each, which in this statistical model is mathematically sufficient and thus representative (Menet et al. 2012; Thaben and Westermark 2016).

## Results

### Demographic data

Human dermal fibroblasts (HDF) were obtained via skin biopsy from volunteers (four men, eight women; 41.50 ± 14.04 years, mean ± SD; BMI: 25.87 ± 5.42 kg/m^2^, mean ± SD). All participants completed the Multiple-Choice Word Test (IQ score: 110.25 ± 9.32, mean ± SD) and Morningness-Eveningness-Questionnaire, German Version (D-MEQ Score: HC: 58.83 ± 8.97, mean ± SD, *p* < 0.01). D-MEQ scores indicated that participants without a neuropsychiatric diagnosis displayed either neutral or morning preferences. The demographic data are presented in Table 2.

**Table 2:** Demographic data

Demographic data	Volunteers without a neuropsychiatric diagnosis (*n* = 12)
Age	41.50 ± 14.04 years
Female	8 (66.7%)
BMI	25.87 ± 5.42
IQ-Score	110.25 ± 9.32
D-MEQ	58.83 ± 8.97
Chronotype	7 (58.3%) neutral 3 (25.0%) moderate morning 2 (16.7%) definite morning

### Actigraphy

The following sleep parameters were measured by actigraphy from participants without a neuropsychiatric diagnosis: mid-sleep on weekend days (3.99 ± 1.57, mean ± SD), mid-sleep on weekdays (3.00 ± 1.59, mean ± SD), social jetlag (0.98 ± 0.85, mean ± SD), sleep efficiency (82.50% ± 9.27%, mean ± SD), WASO (58.90 min. ± 32.40 min., mean ± SD) and total number of wake bouts (25.00 ± 4.86, mean ± SD).

### Cell viability

To establish the optimal remdesivir concentrations, human dermal fibroblasts were incubated with 0.05, 0.50 and 1.00 µM remdesivir for 24 h and compared to negative controls without remdesivir. The viability of cells treated with 0.05 µM remdesivir (84.45 ± 8.62, mean ± SD) and 1.00 µM remdesivir (86.32 ± 5.02, mean ± SD) was decreased compared to control cells without remdesivir (0 µM remdesivir: 89.03 ± 5.66, mean ± SD). The incubation with 0.50 µM remdesivir (89.16 ± 4.88, mean ± SD) did not affect the cell viability.

### Circadian gene expression in human dermal fibroblasts

The expression profiles of circadian genes after incubation with 0.50 µM remdesivir were examined in primary fibroblasts cultured from skin biopsies collected from participants without a neuropsychiatric diagnosis and synchronised with dexamethasone. Cultures without remdesivir were used as negative control.

*Cry1, Per1 and Per2* expression was rhythmic in both groups (CircWave, *p* < 0.001). No rhythmicity was detected for *Clock* in negative controls and cultures incubated with 0.5 µM remdesivir (CircWave, *p* > 0.05). Remdesivir eliminated the rhythmicity of *Bmal1* and *Per3* (CircWave, *p* > 0.05).

**Fig. 1:**
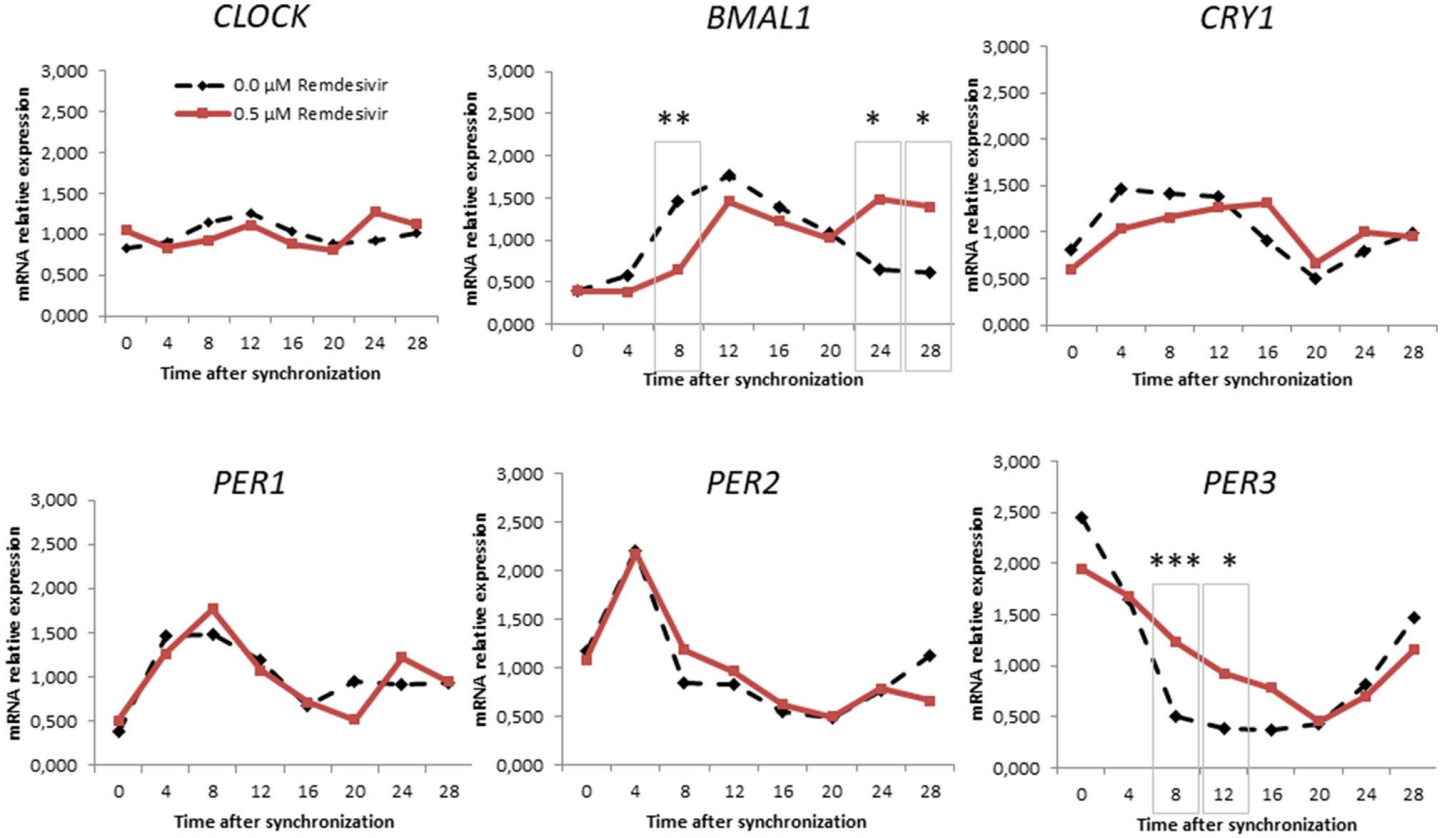
Relative mRNA gene expression of circadian genes in volunteers without a neuropsychiatric diagnosis with 0.0 and 0.5 μm Remdesivir. *p < 0.05, **p < 0.01,*p < *0.001*

Data were normally distributed (Kolmogorov Smirnov). Differences in circadian gene expression levels among study groups were assessed using Student’s’ *t* Test (Fig. 1). Comparing the two groups, significant different expression levels of *Bmal1* at ZT8 (*t*(22) = 3.26, *p* = 0.004), ZT24 (*t*(22) = − 2.66, *p* = 0.015), ZT28 (*t*(20) = − 2.14, *p* = 0.045) and *Per3* at ZT8 (*t*(22) = − 4.27, *p* < 0.001) and ZT12 (*t*(22) = − 2.61, *p* = 0.016) were observed. Remdesivir increased the expression of *Bmal1* at ZT24 and ZT28, as well as the expression of *Per3* at ZT8 and ZT12 compared to the negative control cultures. The expression of *Bmal1* at ZT8 was lower after remdesivir incubation whereas the expression levels at ZT24 and ZT28 was significantly higher compared to the negative control. *Per3* expression level after remdesivir exposure was significantly higher at ZT8 and ZT12 compared to the negative control.

### Chronotype and circadian gene expression

In the current study, 58.3% of participants without a neuropsychiatric diagnosis displayed circadian neutral preference, whereas 25.0% had moderate morning preference, and 16.7% definite morning preference.

**Fig. 2:**
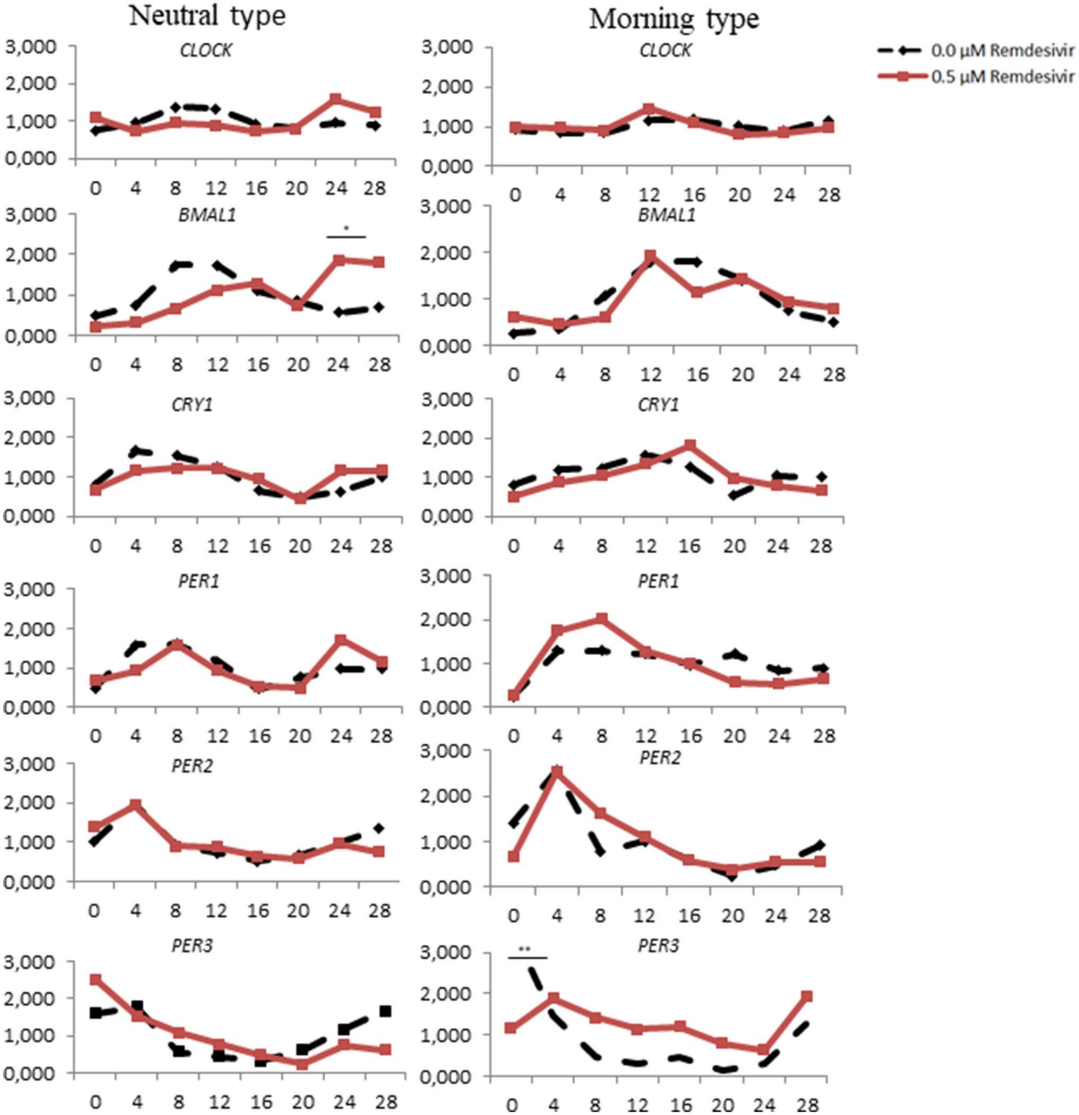
Relative mRNA gene expression of circadian genes in volunteers without a neuropsychiatric diagnosis with 0.0 and 0.5 μm Remdesivir in neutral and morning type. *p < 0.05, **p < 0.01,*p < *0.001*

Differences of circadian gene expression levels among chronotypes were assessed using one-way ANOVA. Statistically significant differences were observed between chronotypes and circadian genes expression for *Per3* (ZT 0, *F* = 7.518, *p* = 0.001; ZT8 *F* = 6.828, *p* = 0.002)*, Bmal1* (ZT8, *F* = 5.107, *p* = 0.009; ZT24, *F* = 4.151, *p* = 0.019) and *Cry1* (ZT16, *F* = 4.216, *p* = 0.018; ZT20 *F* = 3.886, *p* = 0.024). A Bonferroni post hoc correction revealed a significantly decreased expression of *Per3* (*p* = 0.007) at ZT0 in HDFs without remdesivir exposure exhibiting neutral chronotype compared to those with morning chronotype. Also differences in expression between negative controls and HDFs incubated with remdesivir were computed (Fig. 2). The incubation of HDFs with remdesivir significantly lowered the expression of *Per3* (*p* = 0.002) at ZT0 compared to negative controls among participants with morning preferences. Compared to negative control cultures, remdesivir incubation decreased the expression of *Bmal1* at ZT8 in cultures from participants with morning (*p* = 0.023) and neutral (*p* = 0.017) chronotype. In participants with neutral preferences, remdesivir incubation increased the expression of *Bmal1* (*p* = 0.021) at ZT24. In HDFs from participants with morning chronotype, remdesivir also increased the expression of *Per3* at ZT8 (*p* = 0.011) and *Cry1* at ZT16 (*p* = 0.016) compared to negative control cultures from participants with neutral type.

## Discussion

Remdesivir might shorten median recovery time and improve the clinical status of COVID-19 (Pimentel et al. 2020). In an uncontrolled, prospective, open observational study conducted at the Clinic I for Internal Medicine, Cologne University Hospital by Augustin et al., 10-day remdesivir therapy with a target follow-up time of 28 days was carried out (Augustin et al. 2020). The COVID-19 patients received 200 mg IV remdesivir on the first day, followed by 9 days of 100 mg IV remdesivir therapy. 36 of the 53 patients (68%) showed an improvement under remdesivir treatment. Adverse events were reported in 32 of the 53 patients (60%), the most common were elevated liver enzymes, as well as diarrhoea, rash, renal impairment and arterial hypotension. The mortality rate was 13%, with increased risk in patients older than 70 years as well as in patients with a higher serum creatinine level at the start of therapy (Augustin et al. 2020). In May 2020, Beigel et al. designed an adaptive platform to rapidly conduct a series of phase 3, randomized, double-blind, placebo-controlled trials. 1059 patients were randomly assigned to receive either 200 mg loading dose on day 1, followed by 100 mg daily remdesivir for up to nine additional days or placebo for up to 10 days. Beigel et al. observed that remdesivir treatment was superior to placebo in shortening the recovery time in adults hospitalized with COVID-19. Preliminary results indicated that those who received remdesivir had a median recovery time of 11 days as compared with 15 days in those who received placebo. Serious adverse events were reported for 21.1% patients of the remdesivir group and 27.0% patients of the placebo group. Mortality rate by 14 days was 7.1% with remdesivir treatment and 11.9% with placebo (Beigel et al. 2020). Recently, in a study including a cohort of patients hospitalised for severe COVID-19, treatment with compassionate-use remdesivir induced clinical improvement in 68% of patients. Of the 53 patients whose data were analysed, 22 were in the United States, 22 in Europe or Canada, and nine in Japan (Grein et al. 2020).

The risks and benefits of remdesivir in patients with COVID-19 are, however, uncertain. In a meta-analysis, the incidence of all adverse events was higher in the remdesivir group compared to the placebo group (Szarpak et al. 2020). The most common adverse reaction reported are hepatoxicity, cardiac adverse events, gastrointestinal symptoms and respiratory problems (Fan et al. 2020; Gupta et al. 2020; Pimentel et al. 2020).

To the best of our knowledge until now no studies have analysed the influence of remdesivir on circadian rhythm. The results of the present study illustrate that remdesivir impacts on circadian function, particularly, on the *Bmal1* and *Per3* gene expression. Previously, it has been shown that the expression of *Bmal1* and *Period* genes is altered by chronic jet lag on the central and peripheral clocks of mice (Iwamoto et al. 2014). It has been observed, that sleep deprivation in jet lag may relate to a length polymorphism in the *Per3* gene (Cheng et al. 2018; Leocadio-Miguel et al. 2018).

The study shows that *Clock, Cry1, Per1* and *Per2* expression is phase shifted in human dermal fibroblast cultures from participants without a neuropsychiatric diagnosis incubated with remdesivir. Significant different expression levels of *Bmal1* were observed 8, 24 and 28 h after the dexamethasone synchronisation between negative controls and incubation with remdesivir. These results matched the expression of the *Per3* gene, which was significant different at ZT8 and ZT12 in both groups. Remdesivir abolished the rhythmicity of the two genes. We observed a significant differences between chronotypes and circadian gene expression for *Bmal1, Cry1* and *Per3*. In participants with neutral preference, remdesivir incubation increased the expression of *Bmal1* at ZT24 and decreased *Bmal1* expression at ZT8. The same effect for remdesivir was observed in participants with a morning preference. The incubation of HDFs with remdesivir decreased the expression of *Per3* at ZT0 among participants with morning preferences.

It is to mention, that no special cognitive testing was implemented in this study. For further studies a connection between circadian disturbances, cognitive deficits and the effect of medication would be suitable.

In conclusion, human dermal fibroblasts present a suitable model to study circadian rhythms. Circadian rhythms have a profound impact on human health and regulate the pharmacokinetics and efficacy of many therapeutics. For example, dosing-time of drugs regulates the efficacy of viral vaccines (Ray and Reddy 2020). The results of the study suggest that remdesivir medication alters circadian rhythms and circadian gene expression. Remdesivir exposure may alter the sleep quality by inducing a jetlag effect. Circadian rhythmicity is shifted to eveningness similar to the most prevalent chronotype in ADHD.

## Data Availability

Data and material are available.

## Code availability

Actiwatch 2, Philips Respironics, USA; CircWave v. 1.4 software (generated by Dr. Roelof Hut; www.euclock.org); SPSS (IBM Corporation).
